# Transcriptome and Proteome Conjoint Analysis Revealed That Exogenous Sulfur Regulates Glucosinolate Synthesis in Cabbage

**DOI:** 10.3390/plants10102104

**Published:** 2021-10-04

**Authors:** Lushan Li, Hui Zhang, Xiaohong Chai, Shouhui Wei, Shilei Luo, Huiping Wang, Jian Lv, Jihua Yu, Zeci Liu

**Affiliations:** 1 College of Horticulture, Gansu Agricultural University, Lanzhou 730070, China; lils@st.gsau.edu.cn (L.L.); zhangh20210930@163.com (H.Z.); wlj920229@163.com (S.W.); luosl@gsau.edu.cn (S.L.); wanghp@st.gsau.edu.cn (H.W.); lvjian@gsau.edu.cn (J.L.); 2 Panzhihua Academy of Agricultural and Forestry Sciences, Panzhihua 617000, China; 3 College of Grassland Agriculture, Northwest A&F University, Yangling 712100, China; cxhnwasu@163.com; 4 Gansu Provincial Key Laboratory of Aridland Crop Science, Gansu Agricultural University, Lanzhou 730070, China

**Keywords:** sulfur, cabbage, transcriptome, proteome, glucosinolate synthesis

## Abstract

Glucosinolates (GLS) are important anionic secondary metabolites that are rich in thiocyanin in cabbage, *Brassica oleracea* L. var. capitata. GLS are important in food flavor, plant antimicrobial activity, insect resistance, disease resistance, and human anti-cancer effects. Sulfur is an important raw material of GLS, directly affecting their synthesis. However, the mechanism of sulfur regulation of GLS biosynthesis in cabbage is unclear. In the present study, cabbage was treated with sulfur-free Hoagland nutrient solution (control; −S), and normal Hoagland nutrient solution (treatment; +S). Through joint transcriptomic and proteomic analyses, the effect of exogenous S on GLS synthesis was explored. S application induced GLS accumulation; especially, indole glycosides. Transcriptome analysis showed that +S treatment correlated positively with differentially expressed genes and proteins involved in amino acid biosynthesis, carbon metabolism, and plant hormone signal transduction. Compared with −S treatment, the mRNA expression of GLS synthesis genes (CYP, GSTU, UGT, and FMO) and those encoding transcription factors (RLK, MYB, AP2, bHLH, AUX/IAA, and WRKY) were upregulated significantly in the +S group. Combined transcriptome and proteome analysis suggested that the main pathway influenced by S during GLS synthesis in cabbage is amino acid biosynthesis. Moreover, S treatment activated GLS synthesis and accumulation.

## 1. Introduction

Sulfur is a macronutrient required for plant growth [1,2]. It is present widely in proteins, glutathione, plant chelators, thioredoxin, chloroplast membrane lipids, and glucosinolates (GLS) [3]. For many vegetables, sulfur also has an significant ecological role in defense against herbivores and pathogens [4]. The sulfur-containing defense compounds in plants include antimicrobials, thiophenes, S-alkyl cysteine sulfoxides, and GLS. S-alkyl cysteine sulfoxides and GLS also contribute to the flavor and cancer prevention effects of certain vegetables [5]. Cabbage, *Brassica oleracea* L. var. capitata (2n = 18), originated from the Mediterranean to the North Sea, and is a widely planted vegetable worldwide, with high economic and consumption values. According to data released by the Food and Agriculture Organization (FAO), the global cabbage planting area has increased from 282,943 to 286,762 hg/ha from 2016 to 2019. As living standards continue to increase, people’s nutritional quality and health care requirements for vegetable crops are increasing. Cabbage is recognized one of the anti-cancer vegetables, and its anti-cancer activity is mainly derived from thiol glucosides, including isothiocyanate [6]. In recent years, there has been increasing research on cabbage GLS.

GLS are sulfur-rich plant metabolites in the order Brassicales that function in the plant defense against stress and pathogens, and have anti-cancer activities [7]. GLS are soluble in water, and are easily soluble in ethanol, methanol, and acetone. Their degradation products have biological activity, and are currently used in flavor components, cancer-prevention agents, and crop biomitigants. Extensive research on GLS has been performed almost exclusively in Arabidopsis. The chemical structure of GLS usually comprises Sulfonatedoxime, β-D-thio-glucose, and variable side chain R groups derived from amino acids [8]. GLS normally contain two sulfur atoms; therefore, they are good sources of sulfur in plants. GLS acts as a sulfur reservoir, and it could metabolized to supply sulfur for normal growth and development of plants under sulfur deficiency stress [9]. The main factors affecting the GLS content include genetic factors, growth environments, field management, distribution positions, and signal transmission. Jiang et al. [10] studied the effect of leaf surface spraying of methyl jasmonate (MeJA) on the sulfide component of mustard and found that MeJA could not only change the composition of sulfides in mustard, but also their content. Hu et al. [11] treated small cabbage with different concentrations of indoleacetic acid (IAA) and reported that the total GLS content and the aliphatic GLS content of Chinese cabbage increased significantly with the increase in IAA. Further study using genetic markers, chromosome mapping, and other techniques provided in-depth data on the pattern of GLS synthesis in Arabidopsis, especially the synthesis pathway of thioside. The study found that branched-chain amino acid transaminase (BCAT) extends a homologous derivative of methionine and its chain, and its corresponding 2 containing oxyte [12], which demonstrated that BCAT participates in the methionine side chain extension reaction. The methylthioalkylmalate synthase (MAM) gene family encodes proteins that are mainly responsible for the side chain extension reaction of aliphatic GLS originating from methionine [13]. The reaction catalyzed by the proteins encoded by the cytochrome P450 (CYP) gene family is a key step in GLS synthesis, which is mainly responsible for the formation of acetaldoxime. CYP79F1 and CYP79F2 −S the synthesis of aliphatic acetaldoximes [14]. The 2-oxoglutarate-dependent dioxygenases (AOP) gene family encodes proteins that are mainly responsible for the secondary modification of GLS side chains, in which GS-ALK regulates the generation of olefin side chains, GS-OHP controls the synthesis of hydroxyalkyl side chains, and GS-NULL controls the formation of formyl side chains [15]. The GLS metabolic regulation network in Arabidopsis and the regulatory mechanism have been studied more thoroughly, and most genes in the metabolic pathway have been cloned and studied. However, the genes in the cabbage GLS metabolic pathway have rarely been cloned, mainly because of the polyploidy and complexity of its genome. Most GLS synthesis and regulatory genes in cabbage have multiple copies, and there might be differences in the function of the same gene in different species. Therefore, in the present study, we used cabbage as the research object to investigate the transcriptome and proteome, with the aim of enriching our understanding of the synthetic pathway of GLS in cabbage to provide a basis for molecular breeding of GLS in cabbage.

## 2. Results

### 2.1. Content Analysis of GLS under +S and −S Treatment in Cabbage Seedlings

As shown in Table 1, glucosinolate (GLS) components were more abundant in the +S treatment compared with that in the −S treatment, and the contents of each component of GLS in the +S treatment were 2.8 to 6.2 times higher than those of the –S treatment. The content of 4-methoxy increased the most, which was 6.2 times of that in the –S treatment. The second most changed components were 1-methoxyglucose, and brassinol, which increased by 5.15 times. Progesterone and GLS increased by 3.9 and 3.57 times, respectively. The content of glucoside in the two treatments was the highest, followed by sinigrin and 4-hydroxyglucoside. From the total GLS data, the GLS content in +S treatment group was 3.6 times higher than that of the –S treatment group. These results indicated that the addition of S promoted the synthesis of GLS in cabbage.

### 2.2. Transcriptome Sequencing and Correlation Analysis

As shown in Table 2, RNA sequencing of 12 cDNA libraries generated 43.46 Gb of clean data. The Q30 base percentage of each sample was not less than 93.96%, while the highest GC content was 47.96%, and the lowest was 47.60%. Comparison with the clean reads of each sample with the designated reference genome showed that the comparison efficiency was greater than 77.43%. About 74% of the clean reads were mapped uniquely and used for subsequent analysis. For each treatment, we set up three biological replicates to analyze the Pearson correlation coefficients of the expression levels between the different samples. The correlation with the samples in each biological replicate group was 0.8, which indicated good repeatability. The above results demonstrated that the experimental samples and results were considered reliable for further analysis.

#### 2.2.1. Transcriptome Analysis of Exogenous S Treatments in Cabbage

**Figure 1:**
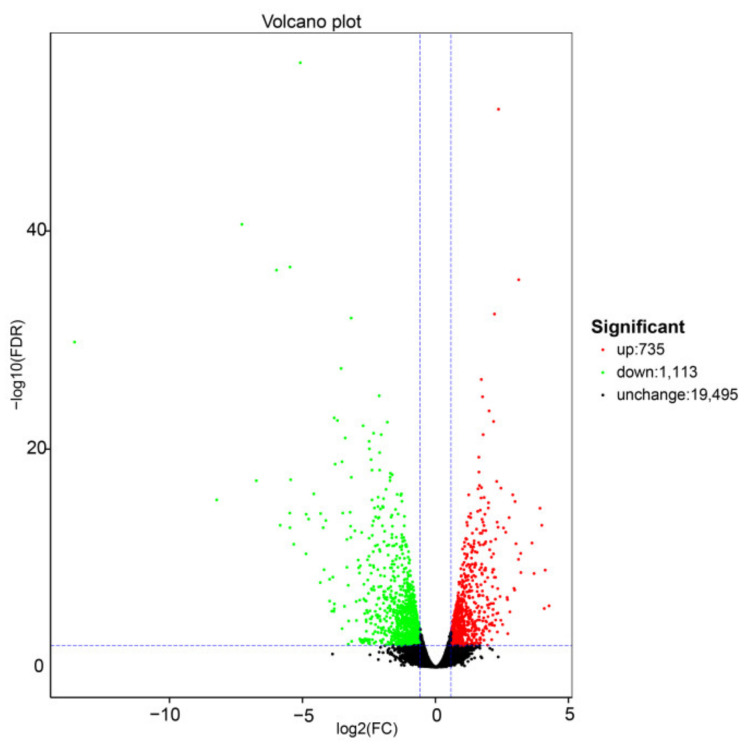
Summary of the number of differentially expressed genes (DEGs) identified by RNA-sequencing analysis and differential expression of Volcano plot of the +S and −S treatments. Each point in the differential expression volcano map represents a gene, and the abscissa represents the logarithm of the differential expression of the gene in the samples. The ordinate represents the negative logarithm of the statistically significant changes in gene expression. The green and red dots represent downregulated and upregulated genes, respectively.

The differential gene expression analysis was performed using the DESeq2 software, and the results are show in Figure 1. DEGs were selected using the false discovery rate (FDR) and a fold-change, with thresholds of FDR < 0.01 and FC >1.5. Volcano plots were used to display the diversity in gene expression levels between the two treatments, which also indicated the statistical noteworthy of the differences. In total, 1848 genes were differentially expressed under +S treatment, 735 were upregulated, and 1113 were downregulated compared with the −S control.

#### 2.2.2. Gene Ontology (GO) Classification Analyses

**Figure 2:**
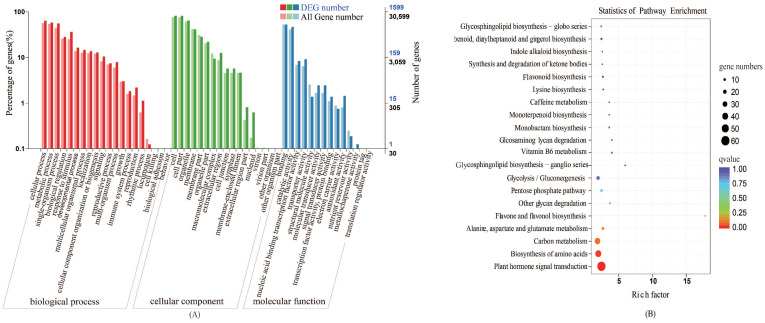
Functional annotation of differentially expressed genes (DEGs) in cabbage under the +S and –S treatment. (**A**) and (**B**) represent gene ontology (GO) annotation and Kyoto Encyclopedia of Genes and Genomes (KEGG) pathway enrichment, respectively. Enrichment factor represents the ratio of genes annotated to the pathway of differential genes to the ratio of all genes annotated to the pathway. Richment factor indicates that the enrichment level of differential genes in the pathway is more significant. *p*-value after the correction for multiple hypothesis testing *q*-value is, the smaller *q*-value represent a significant enrichment of the more reliable. The size of the circle indicates the number of genes enriched in the pathway, and the larger circle, the more genes.

To functionally annotate the differentially expressed genes (DEGs), GO functional annotation was performed using all reference genes as the background. The GO classifications of the 1848 DEGs were annotated, of which 541 were annotated as GO items. The annotated genes were divided into three major functional categories: Biological process (BP), cellular component (CC), and molecular function (MF) (Figure 2). DEGs were mainly annotated in cellular process, metabolic process, single-organism process, cell, cell part organelle, binding, catalytic activity, nucleic acid binding, and transcription factor activity.

#### 2.2.3. Kyoto Encyclopedia of Genes and Genomes (KEGG) Pathway Analyses

KEGG pathway enrichment analysis was performed on the DEGs (Figure 2). We mapped the 1848 DEGs obtained under different sulfur treatments into KEGG pathways, among which 241 DEGs were mapped into 116 metabolic pathways. The main KEGG enrichment categories were: Limonene and pinene degradation, Ribosome, Cutin, suberine and wax biosynthesis, beta-Alanine metabolism, Glycerolipid metabolism, Pentose and glucuronate interconversions, Argine and proline metabolism, etc. These pathways were closely related to the biosynthesis of GLS. Amino acid synthesis and metabolism accounted for the largest proportion of the 20 pathways and the metabolic pathways of hormone precursor substances are significantly different under sulfur-deficient conditions. Therefore, we concluded that biosynthesis of amino acids and plant hormone single transduction under exogenous sulfur treatment play major regulatory roles in the biosynthesis of GLS.

### 2.3. Sulfur Regulates the Synthesis of Cabbage GLS

**Figure 3:**
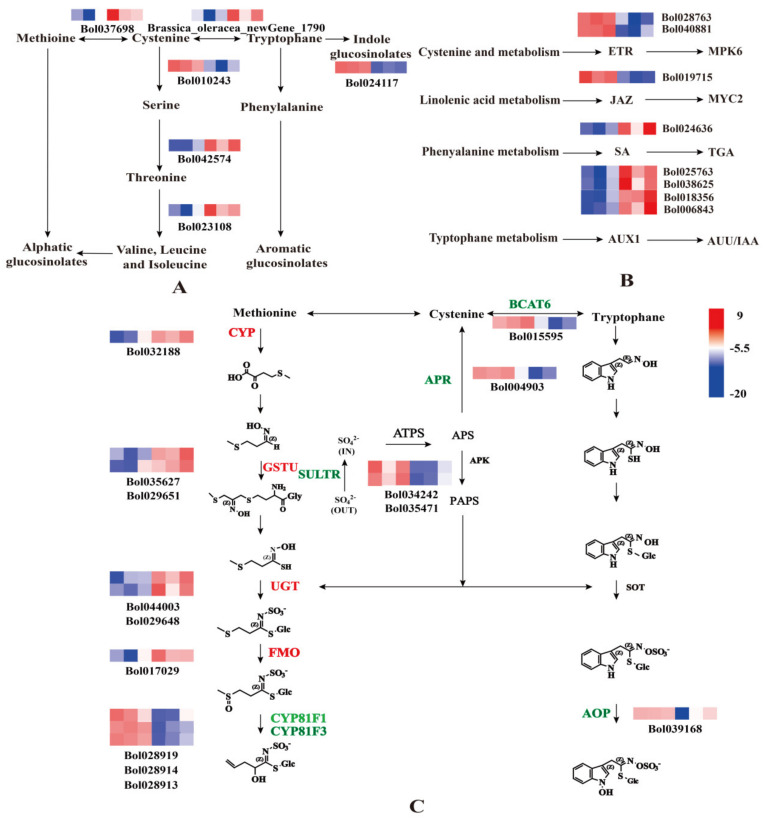
Heat maps depicting the normalized gene expression values, which represent the means ± SD of three biological replicates. Expression values are presented as Fragments Per Kilobase of transcript per Million mapped read (FPKM) normalized log2-transformed counts. (**A**) Heat map of differentially expressed genes (DEGs) involved in the biosynthesis of amino acids. (**B**) Heat map of DEGs involved in the plant hormone signal transduction. (**C**) Heat map of DEGs involved in the synthesis of glucosinolates (GLS). The first three squares of the heat map represent three repetitions of the −S process, and the last three squares represent three repetitions of the +S process. The six genes that regulate Amino acid synthesis are Bol010243(SAT3), new Gene_1790(TSB), Bol037698(METK3), Bol042574(AGT1), Bol023108(OMR1), Bol024117(ADT1). The eight genes that regulate hormone synthesis are Bol018356(IAA3), Bol040881(ERS2), Bol006843(IAA4), Bol028763(ETR2), Bol038615(IAA2), Bol019715(MYC4), Bol024636(TGA4), Bol025763(IAA19). The 14 genes that regulate GLS synthesis are Bol032188(CYP71B26), Bol017029(FMO), Bol029651(GSTU5), Bol035627(GSTU27), Bol029648(UGT71D1), Bol044003(UGT76C1), Bol028919(CYP81F3), Bol015595(BCAT6), Bol028914(CYP81F1), Bol028913(CYP81F1), Bol004903(APR), Bol034242(SULTR4:1), Bol035471(SULTR4:2), and Bol039168(AOP3).

To further explore the role of sulfur in cabbage, we analyzed DEGs that were enriched in biosynthesis of amino acids and plant hormone signal transduction. Exogenous S treatment resulted in the differential expression of 60 genes involved in biosynthesis of amino acids. We focused on the six amino acid synthesis genes that had good sequencing results or are related to GLS synthesis and can regulate GLS synthesis genes (Figure 3A). Cysteine and methionine metabolism (ko00270): gene_Bol010243 and gene_Bol037698; glycine, serine, and threonine metabolism (ko00260): Brassica_oleracea_newGene_1790 and gene_Bol023108; and alanine, aspartate, and glutamate metabolism (ko00250): gene_Bol042574; and phenylalanine, tyrosine, and tryptophan biosynthesis (ko00400): gene_Bol024117. Two of the genes were downregulated and the other four were upregulated. These results implied that the cysteine to serine transformation pathway was inhibited such that more cysteine is converted to methionine, increasing the synthesis of aliphatic GLS. Meanwhile, the pathway of transformation from tryptophan to phenylalanine might also be inhibited, which would be beneficial to the synthesis of indole GLS. These results were consistent with the changes in HPLC determination of GLS contents. There were 49 DEGs that were enriched in plant hormone signal transduction. We focused on eight regulatory genes that are closely related to plant hormone signal transduction; these genes were likely to be related to GLS synthesis (Figure 3B). In summary, S treatment could induce the biosynthesis of amino acids and plant hormone signal transduction.

The GLS biosynthetic pathway has a strong influence on GLS synthesis. Through GO enrichment, and non-redundant (NR) annotation and Swiss Prot annotation, 14 DEGs representing genes involved in the biosynthesis of GLS were identified. Among them, six genes were upregulated and eight genes were downregulated (Figure 3C). Consistent with previous studies [16], the expression levels of sulfate transporters (SULTR) and adenosine-5′-phosohosulfate reductase (APR) genes were downregulated under +S treatment indicating that S inhibited the activation of sulfur transporter genes. Meanwhile the expression levels of GSTU, UGT, and FMO were upregulated, demonstrating that S could promote the synthesis of GLS.

### 2.4. DEG Profiling Validation by qRT-PCR Analysis

To verify the reliability of these DEGs identified by the analysis of the transcriptome using RNA Seq, the expression levels 20 genes were assessed using quantitative real-time revsere transcription PCR (qRT-PCR), including 10 GLS biosynthesis genes and 10 randomly selected genes (five upregulated and five downregulated genes). The qRT-PCR results were consistent with RNA-seq data, which indicated that the gene expression profiles from RNA-Seq were reliable.

### 2.5. Conjoint Analysis of the Proteome after Exogenous S Treatment in Cabbage

To further study the molecular mechanism of S on GLS synthesis in cabbage seedlings, quantitative proteomic techniques were used to analyze the differences in protein abundance between the +S and –S groups. In this study, a 1.2-fold change in protein abundance was used as the criterion to identify differentially abundant proteins. Compared with the −S group, 95 proteins were differentially abundant under +S conditions, with 29 upregulated and 66 downregulated.

#### GO Classification and KEGG Pathway Analyses

**Figure 4:**
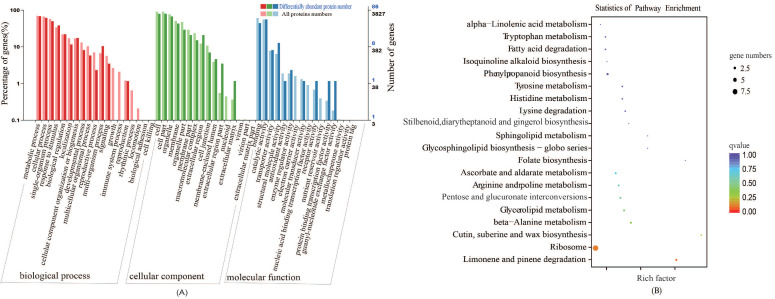
The functional annotation of differentially expressed protein under the +S and –S Treatment. (**A**) and (**B**) represent GO annotation and Kyoto Encyclopedia of Genes and Genomes (KEGG) pathway enrichment, respectively. Enrichment factor represents the ratio of genes annotated to the pathway of differential genes to the ratio of all genes annotated to the pathway. Enrichment factor indicates that the enrichment level of differential genes in the pathway is more significant. *p*-value after the correction for multiple hypothesis testing *q*-value is, the smaller the *q*-value represent a significant enrichment of the more reliable.

Sulfur is an important element in plants. The disulfide bond in proteins plays an irreplaceable role in stabilizing the spatial structure of proteins and regulating their activity. To better reveal the effect of sulfur on the growth of cabbage seedlings, we performed gene ontology (GO) classification analysis on the 95 differentially abundant proteins, which revealed that they are mainly associated with metabolic processes, unicellular processes, cellular processes, binding, and catalytic activity (Figure 4A). According to the Kyoto Encyclopedia of Genes and Genomes (KEGG) enrichment analysis, the differentially abundant proteins were enriched in 38 pathways, mainly including limonene and pinene degradation, Ribosome, Cutin, suberine and wax biosynthesis, beta-alanine metabolism, and glycerolipid metabolism (Figure 4B). We screened the top 20 pathways with the most significant differences. Among the top 20 pathways, we focused on the five pathways of Tryptophan metabolism, tyrosine metabolism, Histidine metabolism, Ascorbate and aldarate metabolism, and beta-alanine metabolism, which involved five different differentially abundant proteins (encoded by gene_Bol005766; gene_Bol024085; gene_Bol036090; gene_Bol038813; and gene_Bol044942). These data indicate that the biosynthesis and metabolism of amino acids after S treatment is one of the main pathways of GLS synthesis.

### 2.6. Association Analysis of the Transcriptome and Proteome

#### 2.6.1. Correlation Analysis of the Transcriptome and Proteome

**Figure 5:**
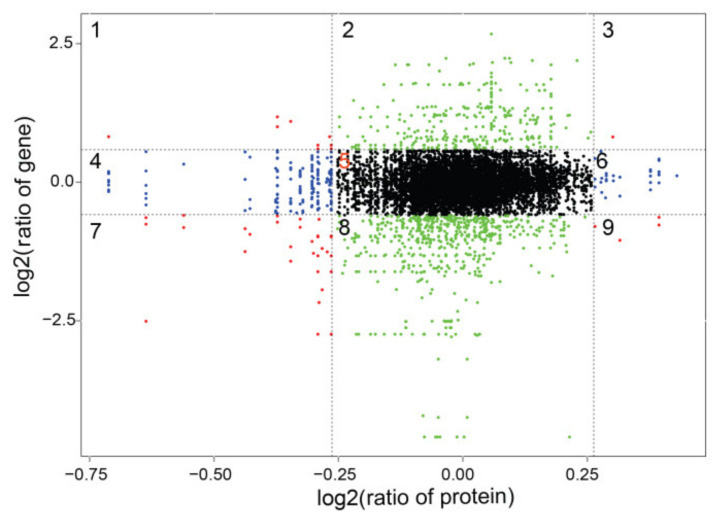
Nine-quadrant diagram of protein and transcriptome expression patterns in different groups. Quadrants 1 and 9 indicate opposite trends of protein and mRNA expression; Quadrant 3 and 7 show the same trend of protein and mRNA expression. Quadrants 2 and 8 indicate no change in protein, but differentially expressed mRNA. Quadrants 4 and 6 indicate no change in differential mRNA and protein expression.

The relationship between mRNA and protein expression levels is complex; therefore, in the present study we analyzed the correlations between transcriptional regulation and protein regulation from the relationship between differentially abundant proteins and mRNA expression trends (quadrants 3 and 7, and quadrants 1 and 9) (Figure 5). A total of 4023 proteins were detected in the experiment, among 95 were differentially abundant proteins. Since the protein abundance in the cell is mainly controlled by the level of translation, there is a significant difference between the protein and mRNA in terms of half-life, synthesis rate, and quantity [17]. We found 347 mRNAs that were associated with the proteomics data, including 308 genes whose differences at the transcriptome level and the protein level did not reached significance, and 39 genes for which the transcripts and proteins levels were significantly different between +S and the control. Among these 39 genes, 7 genes and their encoded proteins were simultaneously upregulated, 31 genes and their encoded proteins were simultaneously downregulated, and 1 gene was upregulated, whereas its encoded protein was downregulated (Table 3).

#### 2.6.2. GO Association Analysis of Proteome and Transcriptome

**Figure 6:**
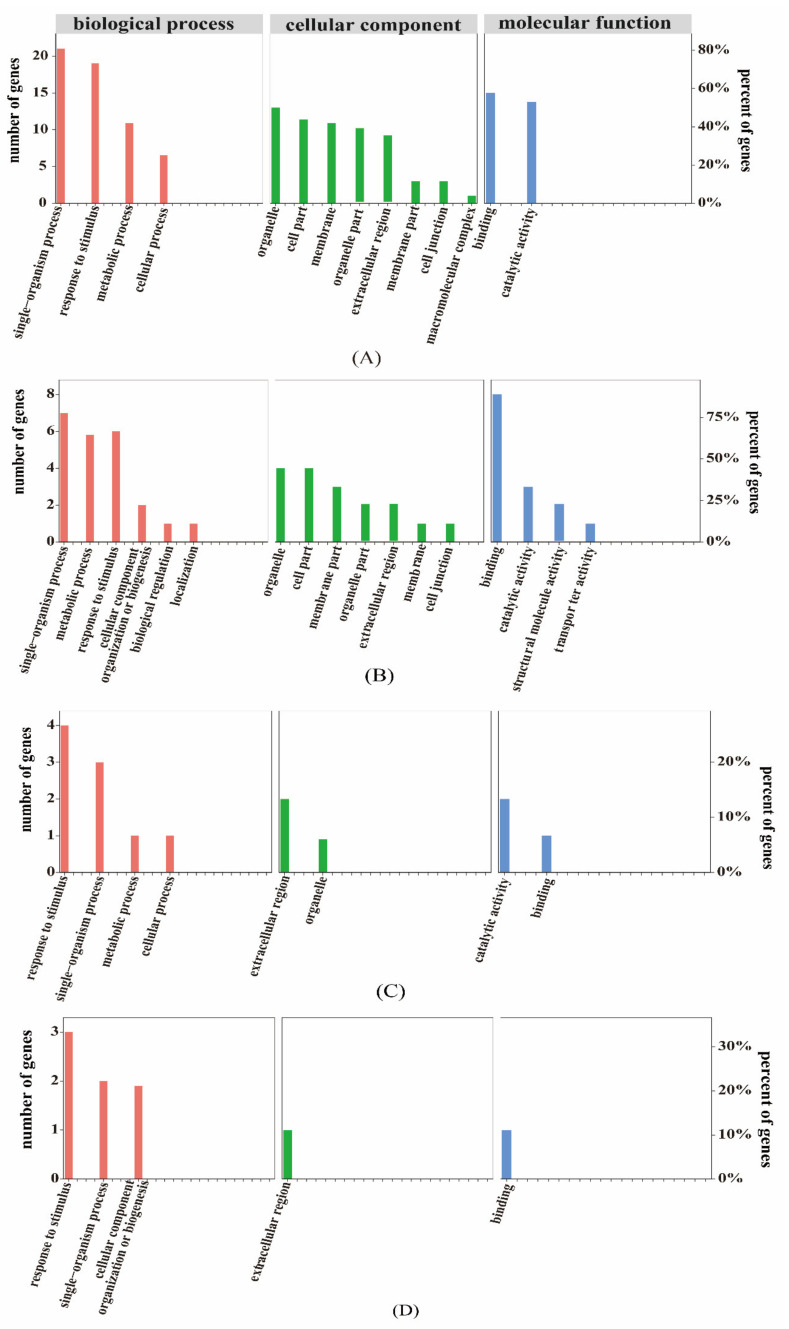
Gene ontology (GO) association analysis of the proteome and transcriptome data. (**A**) represents the expression trends of differentially expressed genes and proteins that are the same as that of the differentially expressed genes in the GO association analysis. (**B**) represents the expression trends of differentially expressed genes and proteins that are opposite to that of the differentially expressed genes the GO association analysis. (**C**) represents the expression trends of differentially expressed genes and proteins that are the same as that of the differentially abundant proteins in the GO association analysis. (**D**) represents the expression trends of differentially expressed genes and proteins that are the same as that of the differentially abundant proteins in the GO association analysis.

GO functional classification was carried out for the differentially abundant proteins whose level was consistent with their encoding mRNA expression trend. Among the cellular components category, they were mainly represented in the organelle subcategory. In the molecular function category, they were mainly associated with catalytic activity and binding. In the biological processes category, they were mainly associated with response to stimulus, single-organism process, metabolic process, and cellular process. The GO function analysis results for differentially abundant proteins whose level showed the opposite trend to the mRNA expression are shown in Figure 6, and are mainly associated with extracellular region in the cell components category. In the molecular function category, they were mainly associated with binding. In the biological process category, were mainly associated with response to stimulus, single-organism process, cellular component organization, and biogenesis. Thus, association to the GO functional analysis of the DEGs and GO analysis of the differentially abundant proteins was significantly enriched in single-organism process, response to stimulus, extracelluar region, and binging.

#### 2.6.3. KEGG Enrichment Analysis of the Proteome and Transcriptome

**Figure 7:**
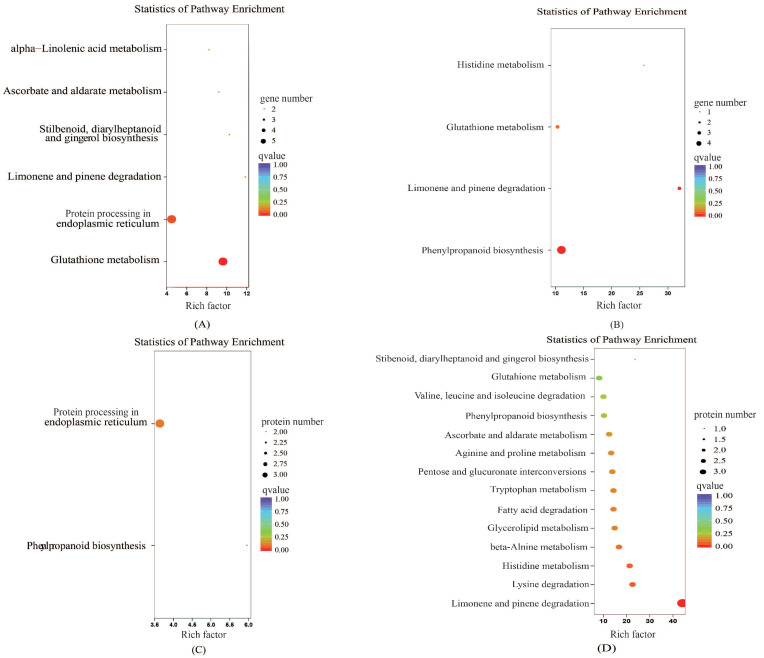
Kyoto Encyclopedia of Genes and Genomes (KEGG) enrichment analysis of the proteome and transcriptome data. (**A**) represents the expression trends of differentially expressed genes and proteins that are the same as that of the differentially expressed genes in the KEGG enrichment analysis. (**B**) represents the expression trends of differentially expressed genes and proteins that are opposite to that of the differentially expressed genes in the KEGG enrichment analysis. (**C**) represents the expression trends of differentially expressed genes and proteins that are the same as that of differentially abundant proteins in the KEGG enrichment analysis. (**D**) represents the expression trends of differentially expressed genes and proteins that are opposite to that of differentially abundant proteins in the KEGG enrichment analysis. Enrichment factor represents the ratio of genes annotated to the pathway of differential genes to the ratio of all genes annotated to the pathway. Enrichment factor indicates that the enrichment level of differential genes in the pathway is more significant. *p*-value after the correction for multiple hypothesis testing *q*-value is, the smaller the *q*-value represent a significant enrichment of the more reliable.

To further explore the biological pathways by which the DEGs and differentially abundant proteins play their roles, KEGG pathway enrichment analysis was conducted. The upregulated differentially abundant proteins were mainly enriched in phenylpropanoid biosynthesis and protein processing in the endoplasmic reticulum, while the genes were mainly enriched in glutathione metabolism, protein processing in the endoplasmic reticulum, and limonene and pinene degradation (Figure 7). The downregulated differentially abundant proteins were mainly enriched in limonene and pinene degradation, and lysine degradation, and the genes were mainly enriched in phenylpropanoid biosynthesis and limonene and pinene degradation.

#### 2.6.4. Associated Differentially Expressed Genes

Through the correlation analysis between the mRNA level and the protein level, 39 proteins were identified in the combined analysis in cabbage seedlings, among which 38 had the same expression trend as the mRNA and 1 had the opposite expression trend. Among the 38 differentially abundant proteins with the same expression trend, 7 were upregulated and the remaining 31 were downregulated. The one differentially abundant protein with the opposite expression trend was upregulated at the mRNA level and downregulated at the protein level. The main pathways involved were: Tryptophan metabolism (ko00380) and valine, leucine, and isoleucine degradation (ko00280). Through the joint analysis of the 39 differentially abundant proteins, we found that they were enriched in 25 different pathways, eight of which were pathways of amino acid biosynthesis (including valine, leucine, and isoleucine degradation; and phenylalanine, tyrosine, and tryptophan biosynthesis); and tryptophan metabolism, beta-alanine degradation, histidine degradation, lysine degradation, and arginine and proline metabolism. These results showed that S treatment mainly affects the synthesis of GLS by modulating the biosynthesis and metabolism of amino acids.

### 2.7. Transcription Factor Analysis of Cabbage Seedlings Treated with and without S

**Figure 8:**
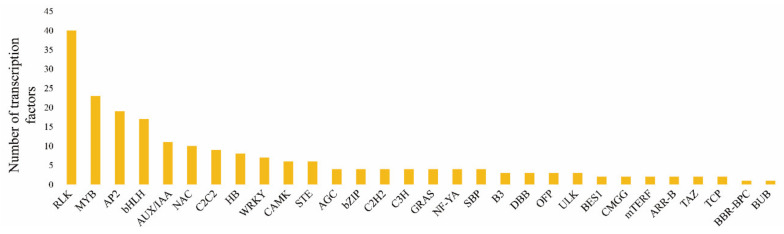
Transcription factor enrichment in +S and −S treatments. The *x*-axis and *y*-axis represent the transcription factors and their amounts in each classification, respectively.

As shown in Figure 8, a large number of transcription factors were activated under +S and –S treatments. Based on transcriptome data, 248 differentially expressed transcription factors were analyzed, among which the most significant were receptor-like kinase (RLK), MYB, AP2, basic helix-loop-helix (bHLH), auxin/indole-3-acetic acid (AUX/IAA), NAC, C2C2, HB, and WRKY. The RLK and MYB type transcription factors had the largest number of activated members, to 40 and 23, respectively. There were 7–19 activated members of the AP2, bHLH, AUX/IAA, NAC, C2C2, HB, and WRKY type transcription factors. We focused on six transcription factors, including RLK, MYB, AP2, bHLH, AUX/IAA, and WRKY. For the RLK group, there were 18 upregulated and 22 downregulated DEGs. For the MYB group, there were 7 upregulated and 16 downregulated DEGs. Similarly, the AP2 group had more downregulated than upregulated DEGs. For the bHLH and AUU/IAA groups, there were more upregulated than downregulated DEGs, indicating that they might play a positive regulatory role in GLS synthesis. All seven DEGs in the WRKY group were downregulated, indicating that they might correlate negatively with GLS synthesis.

## 3. Discussion

In recent years, the incidence of cancer has been increasing. Studies have found that isothiocyanate and other glucosinolates (GLS) degradation products show good defense against cancer. GLS have become the first choice for natural anti-cancer bioactive substances. Hence, investigation GLS has become a research hotspot. Medicinal and edible vegetables with high GLS contents have become a research direction in breeding and cultivation. There are great differences in the types and contents of GLS in different cruciferous crops and in crops grown using different cultivation methods. Therefore, to breed and cultivate cruciferous crops with a high GLS content, many scholars have studied the exogenous substances that control the accumulation of GLS in cruciferous crops. During the growth of Cruciferae, sulfur fertilizer provides a direct source of sulfur for the synthesis of GLS. Inorganic sulfur is absorbed by the root system and transported to the crop body. After the amino acid side chain is extended, the core structure of GLS is improved. The formation and secondary modification of the side chain ultimately generates the variety of GLS. In this study, the content and types of GLS in the leaves of cabbage seedlings treated with sulfur were more than those in the −S group, manifesting that exogenous sulfur has a remarkable effect on the synthesis of GLS. 

As shown in Table 1, compared with the sulfur-deficient treatment, adding sulfur resulted in relatively higher levels of GLS components, mainly gluconapoleiferin. According to a study, gluconapoleiferin has a bitter and pungent taste [18]. Plants assimilate inorganic nitrate into cysteine and other sulfur-containing primary metabolites by absorbing it and using it to synthesize secondary metabolites such as GLS [19,20]. Kim found that in turnip rape, adding sulfur fertilizer increased the GLS content [21,22]. Supplying sulfur to Brassica napus L. increased the proportion of sulfur assimilation to GLS and the proportion of GLS to total sulfur content in vegetative tissues [23]. In the present study, among other GLS components detected, the content of GLS components was higher than that in the control. 4-Methoxyglucobrassicin and 1-Methoxyglucobrassicin have increased by the most multiples. The possible reason is that S treatment regulates the changes of hormone content in plants. In Kushad’s research on GLS content of kale cultivars, Nagoya White and Chidori White had the highest contents of sinigrin and Glucobrassicin [24]. The proportions of these two GLS in Nagoya White and Chidori White were 24% and 30%; and 23% and 37%, respectively. Similar results were obtained in this study. Glucobrassicin had the highest content in our test varieties, comprising more than 38% of the total GLS content. The content of GLS is second only to that of sinigrin, accounting for more than 26% of the total GLS. After S treatment, the ratio of sinigrin and glucoraphanin in total glucosides did not change. This was consistent with the ratios we measured. 

The transcriptome and proteome data showed that +S and −S treatments mainly affected amino acid metabolism, hormone metabolism, and biosynthetic pathways of GLS in cabbage seedlings. Amino acids are precursors of GLS synthesis. Zhai’s results showed that the addition of amino acids could increase the content of GLS in cabbage, and Cys could significantly increase the content of indole GLS [25]. In the present study, we focused on six DEGs in amino acid metabolism. DEGs regulating the synthesis of serine and phenylalanine were downregulated, while those regulating the synthesis of methionine, valine, leucine, isoleucine, and tryptophan were upregulated. Studies have shown that methyl jasmonate can increase the content of indole GLS in cruciferous crops [26]. Li et al. found that the content of GLS increased in the SA signal transduction mutant npr1 [27]. Mikkelsen et al. reported that exogenous application of IAA increased the contents of long-chain aliphatic and indole GLS [28]. Bano also reached the same conclusion after IAA was added during the growth process of rape [29]. Bender proposed that indole acetaldoxime is the junction between IAA and indole GLS in the biosynthesis process [30]. In the present study, among the eight DEGs annotated to be involved in hormone metabolism, five were upregulated (IAA and SA regulatory genes) and three were down-regulated. Therefore, hormones might have significant effects in the synthesis of GLS.

For the biosynthetic pathways of GLS, Ida. summarized glucosinolates synthesis and control genes in the process, including BCAT, MAM, IPMI, CYP, GSTU, SUR, UGT, FMO, AOP, and other members of their gene families [31]. Bednarek showed that amino acids are formed by CYP79 and CYP83 encoded enzymes into isonitro compounds, which interact with glutathione to form S-alkyl hydroxylthioxime [32,33]. Studies have shown that 3C-5C indole GLS are formed under the action of AOP [31]. Under S induction, in the GLS synthesis pathway, CYP771B2, GSTU5, GSTU27, UGT71D1, UGT76C1, and FMO were upregulated, and AOP3, SULTR4:1, SULTR4:2, CYP81F1, and CYP81F3 were downregulated. This is similar to the results of previous studies. The expression levels of these genes were significantly activated during the formation of the core structure of GLS. Our results showed that AOP expression was downregulated, the content of 4-hydroxyglucobrassicin was elevated. Therefore, in cabbage, AOP might play the same role as that in *Arabidopsis* in regulating the synthesis of indole GLS [2,34]. Interestingly, in this study, no differentially expressed genes and proteins of aromatic GLS were detected in either the transcriptome or proteome; meanwhile, no aromatic GLS were detected in the components of GLS by HPLC. This is consistent with the research results in *Arabidopsis* [2,34,35]. Hu et al. also did not find aromatic GLS in the content and composition of cabbage GLS [36]. Therefore, aromatic GLS may not exist in cabbage.

Transcription factors respond to various biotic and abiotic stresses in plants by activating downstream gene expression. Studies have shown that MYB, AP, bHLH, AUX/IAA, and WRKY [37,38,39,40,41] transcription factors are involved in the regulation of the metabolism and synthesis of flavonoids, terpenoids, auxin, alkaloids, and GLS in plants. In rice RLK can regulate WRKY through the MAPK pathway [42,43]. Previous studies have shown that six MYB genes in Arabidopsis thaliana are involved in the regulation of GLS synthesis [44,45]. Yin et al. overexpressed and silenced BoaMYB28 in *Arabidopsis* and the results showed that overexpression caused a significant increase in the aliphatic GLS content in plants, and silencing had the opposite effect [46]. At the same time, the expression levels of MAM1, MAM2, CYP79F1, and CYP79F2, which are 5T5B GLS glucoside synthesis genes, increased significantly [47]. Wittstock’s study showed that the synthesis of indole GLS in cruciferous plants correlated markedly with the synthesis of IAA [48]. Studies have shown that bHLH expression is closely related to the synthesis of GLS [48,49]. Henning’s research showed that bHLH transcription factors interact with MYB28, MYB29, MYB4, MYB51, MYB76, and MYB122 [44]. In addition, MYC4 mutants are expressed to varying degrees in GLS synthesis genes BCAT4, MAM1, CYP79F1, SUR1, UGT, and FMO [50]. According to a previous *Arabidopsis* resistance study, gene CYP81F2 which regulates GLS synthesis in the WRKY18/WRKY40 double mutant was significantly up-regulated [51]. In this study, several classes of transcription factor genes, including RLK, MYB, AP2, bHLH, AUX/IAA, and WRKY, were activated after sulfur treatment. A total of 40 RLKs showed significant differences in expression, which were the most abundant transcription factors. We speculated that RLKs might be closely related to sulfur metabolism and GLS biosynthesis. There were 23, 19, 17, 11, and 7 DEGs, respectively, from the MYB, AP2, bHLH, AUX/IAA, and WRKY classes of transcription factor genes. There were fewer upregulated MYB and AP2 DEGs than downregulated DEGs. This was not consistent with previous studies. The possible reason is that MYB and AP2 are large gene families, and the genes regulating GLS synthesis only account for a small part of them, which might have resulted in more downregulated genes. There were 10 upregulated AUU/IAA genes, and only one downregulated AUU/IAA, gene, which indicated that AUU/IAA might correlate positively with the synthesis of GLS. In our study, the number of positively regulated genes (13) was greater than the number of negatively regulated genes (4) in the bHLH group. This suggested that bHLH transcription factors mostly play a positive role in the synthesis of GLS. All seven WRKY genes were significantly downregulated, thus WRKY transcription factors likely have a negative regulatory effect on the synthesis of GLS. Although there are few reports on the regulation of GLS synthesis by WRKY transcription factors in cruciferous vegetables, our results suggested that WRKY transcription factors regulate the biosynthesis of GLS in cabbage seedlings. Taken together, the results suggest that transcription factors from the RLK, MYB, AP2, bHLH, AUX/IAA, and WRKY groups might are involved in the regulation of GLS synthesis. However, the specific regulatory process of these transcription factors on the synthesis of GLS in cabbage is still unclear, and further verification is needed.

## 4. Materials and Methods

### 4.1. Plant Materials

Cabbage (*Brassica oleracea* L.) plants were grown in a greenhouse in Gansu Agricultural University from August to September 2020. The cabbage seeds are sowed in the seedling bowl, and they are allowed to grow in the seedling bowl for ten days without any treatment, after which the seedlings were grown in 1/8 nutrient solution. The seedlings were planted at two plants per 400 mL pot. Water Hoagland nutrient solution every five days. The treatments consisted of two S supply levels (2 mM, S sufficiency and 0 mM, S deficiency). Except for sulfur, the rest of the nutrient solution had the same composition in the two groups. The experiment was set up in three replicates, and 30 pots of cabbage were planted in each replicate. The plants were harvested 30 days after treatment initiation. The leaves were immediately frozen in liquid nitrogen and freeze-dried.

### 4.2. Extraction and Identification of Glucosinolates

Glucosinolates (GLS) were extracted according to the methods of Yang et al. [52], with some modification. First, 0.25g freeze-dried sample for each replicate placed in a centrifuge tube. Four milliliters of 75 °C preheated 70% methanol was added, followed by Glucotropaeolin as an internal standard (200 μL, 5 mM) and the tube was placed in a 75 °C water bath for 10 min. The sample was centrifuged at 5000× *g* for 10 min, and the supernatant was collected into a 10 mL volumetric flask. The precipitate was extracted twice using the above method, and the two supernatants were added to the original volumetric flask, and diluted to 10 mL with methanol. The solution was filtered through filter paper, and then 5 mL of the filtrate was added to a DEAE Sephadex A25 solid phase extraction column. After all the filtrate was allowed to flow, 6 mL of ultrapure water was added twice, and finally 250 µL of sulfates was added, and enzymatic hydrolysis was allowed to proceed at 30 °C for 12 h. Elution was then performed using 5 mL of ultrapure water, and the eluate was filtered through a 0.45 µm filter membrane to obtain the GLS extract. GLS were determined using high performance liquid chromatography (HPLC). The injection volume was 20 µL, the chromatographic column was a C18 reversed phase column (250 lines, 5 µm, Bischoff, Leonberg, Germany), the column temperature was 30 °C, the mobile phase was water and acetonitrile, the gradient was 0–45 min, the acetonitrile gradient was 0–20%, the detection wavelength was 229 nm, and the flow rate was 1 mL/min. The GLS content was calculated based on the internal standard value.

### 4.3. RNA Extraction and cDNA Library Construction

First, 0.1g sample for each replicate is ground in a mortar containing liquid nitrogen and placed in a centrifuge tube. Then, 1 mL of TRizol reagent (Thermo Fisher Scientific, Waltham, MA, USA) was added and vortexed immediately at 25 °C; next, chloroform was added and centrifuged, the supernatant was retained, and isopropyl alcohol was added, incubated for 10 min, and centrifuged again. One milliliter of 75% ethanol was added and mixed, and the tube was centrifuged again. Finally, the supernatant was discarded, and the pellet was dried and resuspended in a suitable amount of RNase-free water. The RNA concentration was detected using a Nanodrop spectrophotometer (Nanodrop Technologies, Wilmington, DE, USA). A 3 µg sample of total RNA was taken from each sample as the starting material to construct the transcriptome sequencing library, which was performed according to the instructions of the NEBNext ^®^ UltraTM RNA Library Prep Kit for Illumina Step one plus (E7530L, NEB, Ipswich, MA, USA). After library construction, an Agilent 2100 instrument and a real-time PCR System were used to check the library (Agilent Technologies, Santa Clara, CA, USA). Clusters were generated on the cBot using HiSeq PE Cluster Kit v4-cBot-HS (Illumina, San Diego, CA, USA). The double-ended sequencing program (PE) was run on a HiSeq4000 sequencing platform. Clean Data was obtained by filtering the data with double-ended sequencing reads of 150 bp. Bowtie V1.0.1 (Johns Hopkins University) was used to build the reference genome library, and then the clean data were compared with the reference genome using TopHat V2.0.12 (Johns Hopkins University Center for Computational Biology) to obtain the location information of the gene on the reference genome, and the annotation information for the associated genes. The clean reads were then subjected to further analysis and annotation.

### 4.4. DEG Screening and Functional Analysis

DEG Seq V1.18.0 (Bioconductor Open Source Software for Bioinformatics) was used to analyze the DEGs on the basis of their expression levels, and the *p*-value obtained was corrected to control for false positives according to Benjamin and Hochberg methods. After correction, the FDR threshold was set as 0.05. The Log2 (Fold Change) threshold was set as 1.5. After screening and identifying the DEGs, GO, KEGG, NR, Pram, and Swiss-PROT analyses were used to identify the functions of the DEGs.

### 4.5. Proteome Extraction and Profiling

Select the third leaf from the growth point of the cabbage from the inside to the outside. The plant tissue samples were ground under liquid nitrogen and transferred to A 1.5 mL centrifuge tube. Then, 4 mL PE Solution A, 4ml PE Solution B and 4 mL PE Solution C were successively added. The supernatant was discarded overnight and centrifuged for 15 min. Acetone was added to the precipitate and centrifuged for 5 min to collect the precipitate. Then, acetone was added again to collect the precipitate and 4 mL PE Solution D was added to obtain the supernatant as a protein solution. Isopropyl alcohol (70 μL) was added to each tube, vortexed for 1 min, centrifuged and restored the labeling reagent to room temperature. The labeling reagent was added to the pre weighed peptide segments (about 100 μg) of each sample, and different samples were labeled with isotopes of different sizes. Protein samples were labeled as 126, 127N, 128C, 129N, 130C, 131. After labeling, 10 μg samples were mixed and then analyzed using high precision LC/MS spectrometry (Thermo fisher—Q—Exactive—Orbitrap). After scanning the mass spectrometry signal, the general layout was completed, and then converted it to a MGF file using Mascot software (Mascot 2.3.01) for quantitative retrieval and qualitative analysis. The relative quantification of peptide was calculated according to the relative abundance of ions, and the relative quantification of protein was calculated according to the relative quantification of peptide. Differences in protein screening criteria: the same protein in a relative to the other group of ratio greater than 1.2 times (up) or less than 1.2 times (down), and *p* < 0.05. Through a searchable database of this experiment transcriptome database, to GO ultimately identified protein function. The database search software is Proteome Discover 2.4. [53,54,55].

### 4.6. Validation of DEGs Using qRT-PCR

According to the nucleic acid sequences of the DEGs, primers for quantitative real-time PCR were designed by PrimerPremier6 software (Premier Biosoft, Palo Alto, CA, USA), and synthesized by the Lanzhou branch of Beijing Kinco Biotechnology Co., Ltd. The cabbage actin gene (ACTIN) was used as the internal reference gene. The mRNA of cabbage leaves was extracted for first-strand cDNA synthesis using Superscript^®^IIIRT (Invitrogen, Waltham, MA, USA). Trans Start ^®^ Top Green qPCR Super Mix (AQ131) from Transgen Biotechnology Co, Ltd. (Beijing, China) was used for qPCR detection. The reaction system consisted of 10 µL of qPCR Mix, 1 µL of upstream primer (10 µmol l^−1^), 1 µL of downstream primer (10 µmol l^−1^) and 2 µL of cDNA template and sufficient ddH_2_O added to the mixture for a total volume of 20 µL. The qPCR reaction and data collection were performed on a Roche Light Cycler 96 instrument (Roche, Basel Switzerland). The qPCR reaction conditions were 95 °C for 30 s; followed by 40 cycles of 95 °C for 5 s, 60 °C for 30 s, and 72 °C for 10 s. After the reaction was completed, the specificity of the reaction product was analyzed using melting curve analysis, and the PCR products were sequenced to determine the specificity of the reaction. The relative gene expression was calculated using the 2^−^^△△Ct^ method [56].

## 5. Conclusions

The present study confirmed that S can promote the accumulation of glucosinolates in cabbage seedlings. In addition, on the basis of transcriptomic analysis, that found that most of the key genes related to amino acid metabolism, hormone metabolism, and biosynthesis of GLS responded to S treatment in cabbage. Through proteome analysis and proteome and transcriptome conjoint analysis, we found that the synthesis and metabolism of amino acids affect the biosynthesis of GLS. Several possible transcription factors related to GLS synthesis, such as RLK, BHLH, AP2, MYB, AUX/IAA, and WRKY, were identified. The results of the present study increase our understanding of the mechanism GLS synthesis under S treatment at the molecular level.

## Figures and Tables

**Table 1:** Effect of sulfur supply on the composition and content of glucosinolates.

Treatment	Alphatic Glucosinolates/μmol·g-1 (DW)			Indole Glucosinolates/μmol·g-1 (DW)			
	Progoitrin	sinigrin	gluconapoleiferin	4-Hydroxyglucobrassicin	Glucobrassicin	4-Methoxyglucobrassicin	1-Methoxyglucobrassicin
+S	1.25± 0.10 a	6.17 ± 0.98 a	0.28 ± 0.1 a	3.79 ± 0.51 a	9.01 ± 1.29 a	1.37 ± 0.2 a	1.82 ± 0.24 a
−S	0.32 ± 0.03 b	1.82 ± 0.67 b	0 b	1.35 ± 0.48 b	2.5 2± 0.86 b	0.22 ± 0.05 b	0.33 ± 0.1 b

Date are the means ± standard deviation of three replicates. Mean followed by same letter were not significantly different (*p* > 0.05).

**Table 2:** Sequencing Data Statistics.

Treatment	Total Reads	Clean Reads	Q30	Map Read	GC Content
+S	42,384,400	2,119,200	94.26%	32,970,027 (77.79%)	47.67%
	58,336,300	29,168,150	94.37%	45,400,252 (77.83%)	47.96%
	47,468,320	23,734,160	94.12%	36,767,459 (77.46%)	47.65%
−S	44,430,344	22,215,172	93.96%	34,403,152 (77.43%)	47.60%
	58,451,572	29,225,786	94.70%	58,451,519 (77.86%)	47.59%
	40,308,466	20,154,233	94.83%	31,383,916 (77.86%)	47.62%

Total reads: The number of clean reads, calculated on a single-ended basis. Clean reads: Total number of pair-end reads in the clean data. Map reads: The number of reads mapped to the reference genome and the percentage of reads in the clean reads. GC content: Clean data GC content, that is, the percentage of bases G and C in the total bases in the clean data Q30%: The percentage of bases whose clean data mass value was greater than or equal to 30.

**Table 3:** Common differentially expressed genes between the proteome and the transcriptome.

Protein Name	Regulation	Gene Name	Regulation	GO Annotation	KEGG Annotation
gene_Bol013541	down	gene_Bol013541	down	Molecular Function	K14709
gene_Bol009168	down	gene_Bol009168	down	Molecular Function	K03152
gene_Bol027549	down	gene_Bol027549	down	Molecular Function	K09580
Bo_newGene_2279	down	Bo_newGene_2279	down	Molecular Function	-
gene_Bol028304	down	gene_Bol028304	down	Cellular Component	-
gene_Bol014966	down	gene_Bol014966	down	-	-
gene_Bol016517	down	gene_Bol016517	down	Molecular Function	K00799
gene_Bol040974	down	gene_Bol040974	down	Molecular Function	K14709
gene_Bol031335	down	gene_Bol031335	down	Molecular Function	K08245
gene_Bol008941	down	gene_Bol008941	down	Biological Process	K01366
gene_Bol016209	down	gene_Bol016209	down	Molecular Function	K12657
gene_Bol039726	down	gene_Bol039726	down	Cellular Component	K13496
Bo_newGene_299	down	Bo_newGene_299	down	-	-
gene_Bol028307	down	gene_Bol028307	down	Cellular Component	-
gene_Bol029650	down	gene_Bol029650	down	Biological Process	K01657
gene_Bol029161	down	gene_Bol029161	down	Cellular Component	K10525
gene_Bol041097	down	gene_Bol041097	down	Cellular Component	-
gene_Bol021589	down	gene_Bol021589	down	Molecular Function	-
gene_Bol025300	down	gene_Bol025300	down	Molecular Function	-
gene_Bol043996	down	gene_Bol043996	down	Molecular Function	-
gene_Bol022426	down	gene_Bol022426	down	Biological Process	K01057
gene_Bol022099	down	gene_Bol022099	down	Cellular Component	-
gene_Bol028238	down	gene_Bol028238	down	Cellular Component	-
gene_Bol004625	down	gene_Bol004625	down	Molecular Function	K00799
gene_Bol024581	down	gene_Bol024581	down	Cellular Component	K07407
gene_Bol033864	down	gene_Bol033864	down	Molecular Function	-
gene_Bol025335	down	gene_Bol025335	down	-	-
gene_Bol036090	down	gene_Bol036090	down	Molecular Function	K00423
gene_Bol038487	down	gene_Bol038487	down	Cellular Component	-
gene_Bol037300	down	gene_Bol037300	down	-	-
gene_Bol031446	down	gene_Bol031446	down	Molecular Function	-
gene_Bol024085	down	gene_Bol024085	up	Molecular Function	K00128
gene_Bol042093	up	gene_Bol042093	up	Molecular Function	K09872
gene_Bol011158	up	gene_Bol011158	up	Cellular Component	-
gene_Bol029420	up	gene_Bol029420	up	Molecular Function	K00008
gene_Bol033052	up	gene_Bol033052	up	Cellular Component	-
gene_Bol040405	up	gene_Bol040405	up	Molecular Function	-
gene_Bol033653	up	gene_Bol033653	up	Molecular Function	K08235
gene_Bol031534	up	gene_Bol031534	up	Cellular Component	-

The first and third columns represent the differentially expressed proteins and genes in the joint analysis. The second and fourth columns represent the differentially expressed proteins and genes expression trends in the joint analysis. The fifth and sixth columns represent the GO and KEGG annotations of the differentially expressed proteins and genes in the joint analysis.

## Data Availability

The original transcriptome data can be found at the following website: https://www.ncbi.nlm.nih.gov/sra/PRJNA745091. Raw proteome data can be found at the following website: https://www.ebi.ac.uk/pride/profile/reviewer_pxd027363.
